# Genetic variation in the 3’ untranslated region of dengue virus serotype 3 strains isolated from mosquitoes and humans in Brazil

**DOI:** 10.1186/1743-422X-10-3

**Published:** 2013-01-02

**Authors:** Márcia Gonçalves de Castro, Fernanda Bruycker de Nogueira, Rita Maria Ribeiro Nogueira, Ricardo Lourenço-de-Oliveira, Flávia Barreto dos Santos

**Affiliations:** 1 Laboratório de Trasmissores de Hematozoários, Av. Brasil 4365, Rio de Janeiro, RJ, 21045-900, Brasil; 2 Laboratório de Flavivírus, Instituto Oswaldo Cruz, Fundação Oswaldo Cruz, Av. Brasil 4365, Manguinhos, Rio de Janeiro, RJ, 21045-900, Brasil

**Keywords:** Dengue virus type 3, Aedes aegypti, 3′UTR

## Abstract

**Summary:**

**Background**

Dengue, a mosquito-borne viral infection caused by one of the four dengue virus (DENV) serotypes (DENV-1 to 4), replicate alternately on the mosquito vector and human host and are responsible for infections throughout tropical and subtropical regions of the world. In Brazil, the disease has become a major public health problem and the introduction of DENV-3 in 2000 in Rio de Janeiro (RJ) was associated with severe dengue epidemics. The potential emergence of strains associated with severe disease highlights the need for the surveillance of DENV in human host and vectors.

**Methods**

Aiming to contribute for DENV phylogenetic and vector-virus-human host studies, we sequenced the entire genome of one DENV-3 isolated from naturally infected *Aedes aegypti* from RJ in 2001 and characterized the 3’ UTR from strains isolated from mosquitoes and humans. Mosquitoes were pooled and submitted to virus isolation in *Ae. albopictus* C6/36 cells and the infecting serotype was identified by immunofluorescence using type-specific monoclonal antibody. Sequence analysis was performed using BioEdit software, the multiple alignments were performed using CLUSTAL W and the phylogenetic analysis by MEGA 5, using the Neighbor-joining method. Secondary structure prediction was performed by using the MFOLD program.

**Results**

Exclusive substitutions and a substitution leading to a stop codon on the NS5 gene were observed in the DENV-3 isolated from a naturally infected *Ae. aegypti* and fully sequenced. As an 8- nucleotides deletion was observed within the 11- nucleotides (nts) insertion on the variable region (VR) from the 3′UTR in this isolate, we further sequenced other DENV-3 from both mosquitoes and humans. The majority of DENV-3 from RJ analyzed were characterized by the 11-nts insertion in the VR of the 3′UTR, despite the observation of strains carrying the 8-nts deletion. The latter presented similar secondary structures, however not all strains presenting the 11-nts insertion were similar in the predicted secondary structure.

**Conclusions**

The phylogeny based on the analysis of the complete genome and 3′UTR characterized the DENV-3 isolated from both vector and human host as belonging to Genotype III (GIII), despite the differences observed on the 3’ UTR. Further studies are needed to address the role of those mutations in the transmission of the different viral populations and vector competence.

## Background

Dengue is a mosquito-borne viral infection caused by one of the four dengue virus serotypes (DENV-1 to 4), belonging to genus *Flavivirus*, family *Flaviviridae.* The viruses replicate alternately on the mosquito vector, mainly (*Ae. aegypti*) and human host and are responsible for infections throughout tropical and subtropical regions of the world [1,2].

The rapid global spread of the four DENV serotypes in the last 50 years resulted in the dispersal of genotypes associated with increased disease severity [3]. In Brazil, dengue has been a major public health problem since DENV-1 introduction and spread in 1986 [4], however the introduction of the genotype III of DENV-3, in December 2000, in Nova Iguaçu, State of Rio de Janeiro (RJ), caused one of the most severe epidemics reported in the country in 2002 [5-7]. Despite the co-circulation of DENV-1, DENV-2 and DENV-3 in that area, DENV-3 was the only serotype detected in pools of *Ae. aegypti* during an entomological surveillance performed [8].

Sequencing of distinct DENV genomic regions has identified five genotypes for DENV-3: Genotypes I to III (GI to GIII) which are responsible for most DENV-3 human infections and have been associated with both dengue fever (DF) and dengue haemorrhagic fever (DHF) epidemics in Southeast Asia, Indian Subcontinent, South Pacific and East Africa and Americas, and Genotypes IV and V (GIV and GV) which were not associated with DHF epidemics and are only represented by few early sequences from Americas, South Pacific and Asia [9-13].

The DENV genome is composed by a positive single-stranded RNA of approximately 11 kb in length with an open reading frame encoding for the viral polyprotein, which is cleaved into three structural proteins (C, prM and E) and seven non-structural proteins (NS1, NS2A, NS2B, NS3, NS4 and NS5) flanked by 5′ and 3′ untranslated regions (UTRs) of about 100 and 400 nucleotides, respectively [1]. The flaviviruses UTRs are predicted to form secondary stem-loop (SL) structures, which are highly conserved and play a role in viral replication [14-18].

According to predicted secondary structures, the DENV 3′UTR can be divided into three domains [18]. The domain I, which is located immediately after the NS5 stop codon, is considered the most variable region (VR) within the viral 3′UTR, as it shows large heterogeneity in both length and nucleotide sequences [19-21]. Mutations and deletions within these regions may alter infectivity and reduce efficiency of viral replication [22,23] and differences between strains in these regions may correlate with DENV virulence and pathogenicity [24-27]. Furthermore, deletions and nucleotide variations were also described in the VR within the same serotype [28-30]. Domain II is of moderate conservation, comprising several hairpins motifs and where conserved sequence (CS2) and repeated CS2 (RCS2) are present. Domain III is the most conserved region of the 3′UTR with CS1 followed by a terminal stem-loop (3′SL) [18].

Here, aiming to contribute for the studies on human host-virus-vector interactions, we fully sequenced the genome of one DENV-3 isolated from naturally infected field-caught mosquitoes in RJ and characterized the viral 3′UTR in comparison to other sequenced DENV-3 isolated from naturally infected mosquitoes and human hosts.

## Material and Methods

### Ethical Statement

All human DENV-3 strains belong to a previously gathered collection from the Laboratory of Flavivirus, IOC/FIOCRUZ, RJ, Brazil obtained from acute phase human serum through the passive surveillance system from an ongoing Project approved by resolution number CSN196/96 from the Oswaldo Cruz Foundation Ethical Committee in Research (CEP 274/05), Ministry of Health, Brazil.

*Ae. aegypti* examined in this study were collected by the staff of the Dengue Control State Program for the determination of house infestation index, virological and entomological surveillance. No special permission or written consent is required for house entrance for mosquito collection and larval site treatment.

### Viral strains

The DENV-3 strains isolated from *Ae. aegypti* adult mosquitoes (*n=*4) and human hosts (*n=*10) naturally infected in RJ were collected from epidemics occurred from 2001 to 2008. The first Brazilian DENV-3 strain (BR74886/02) isolated from a human fatal case fully sequenced [31] was used for comparison purposes and detailed information on the strains is provided on Table 1.

**Table 1:** Brazilian DENV-3 isolated from naturally infected vectors and human cases analyzed in this study

**Strain**	Origin State*	**Year of isolation**	**Source**	**GenBank accession number**	**Sequence analyzed**	**Reference**
BR73354/01	RJ	2001	Mosquitoes	FJ177308	Complete genome	This study
BR73356/01	RJ	2001	Mosquitoes	JN383345	3′UTR	This study
BR73636/01	RJ	2001	Mosquitoes	JN383346	3′UTR	This study
BR81200/06	RJ	2006	Mosquitoes	JN383344	3′UTR	This study
BR70562/01	RJ	2002	Human serum	JN380902	3′UTR	This study
BR74792/02	RJ	2002	Human serum	JN380899	3′UTR	This study
BR74916/02	RJ	2002	Human serum	JN380901	3′UTR	This study
BR74947/02	RJ	2002	Human serum	JN380904	3′UTR	This study
BR77515/03	RJ	2003	Human serum	JN380900	3′UTR	This study
BR78969/04	RJ	2004	Human serum	JN380905	3′UTR	This study
BR80740/05	RJ	2005	Human serum	JN380906	3′UTR	This study
BR80996/06	RJ	2006	Human serum	JN380903	3′UTR	This study
BR83904/07	RJ	2007	Human serum	JN380898	3′UTR	This study
BR072/08	RJ	2008	Human serum	JN380907	3′UTR	This study
BR74886/02	RJ	2002	Human liver	AY679147	Complete genome	Miagostovich et al., 2006

*RJ: Rio de Janeiro.

### DENV-3 human cases

From 2001 to 2008, the Laboratory of Flavivirus, as a Regional Reference Laboratory for the Brazilian Ministry of Health, received a total of 16,185 dengue suspected cases for routine diagnosis. Virus isolation was attempted in 9,405 cases and DENV-3 was the infecting serotype in 52.8% of the positive isolates. The samples analyzed in this study were chosen randomly and representative of each year, during and after the 2002 epidemic in RJ.

### DENV-3 entomological surveillances

The three DENV-3 strains isolated (BR73354/01, BR73356/01 and BR73636/01) in 2001 from naturally infected *Ae. aegypti* adult mosquitoes used in this study were collected during an entomological survey performed in 35 districts of Nova Iguaçu, RJ, from July 2000 to June 2001. The other DENV-3 strain (BR81200/06) was isolated from naturally infected *Ae. aegypti* adult mosquitoes collected during an entomological survey conducted on 7 districts with different infestation index, randomly chosen in the municipality of Rio de Janeiro, RJ, from March 2005 to February 2006. Briefly, adult mosquitoes were collected twice a week, alternately in the morning and in the afternoon with manual and battery backpack aspirators and with nets, both indoor and in the yards and gardens, close to the dwellings. Mosquitoes were identified using a key as previously described [32], pooled according to gender, date, district of collection and stored in liquid nitrogen at the same day of collection. A total of 503 *Ae. aegypti* mosquitoes (352 females and 151 males) collected in 2000–2001 and 874 *Ae. aegypti* females collected in 2005–2006 were pooled (74 pools of 9–17 mosquitoes/pool in 2000–2001 and 27 pools of 2–10 mosquitoes/pool, jn 2005–2006) and all pools were submitted for virus isolation. Only positive pools were submitted to RT-PCR. for DENV serotype confirmation.

### Preparation of vectors

Mosquitoes’ pools were macerated in 1 ml of Leibovitz L-15 medium (Sigma) plus antibiotics (penicillin-streptomycin, 10,000 units - Invitrogen) and centrifuged (6,000 rpm at 4°C for 30 min). Supernatant was transferred to an Eppendorf tube containing 100 mL of streptomycin / fungizone and penicillin, kept in an ice bath for 1 hour and centrifuged (3,000 rpm at 4°C for 15 min). Supernatant was transferred to an Eppendorf tube containing 0.3ml of fetal calf serum (Invitrogen) and frozen (−70°C).

### Virus isolation

Virus isolation was performed by inoculation into monolayers of C6/36 *Aedes albopictus* cells [33] in Leibovitz L-15 medium (Sigma) supplemented with 2% fetal calf serum (Invitrogen) and 0.2 mM of nonessential amino acids (Invitrogen). Cells were incubated at 28°C for 5 to 7 days and observed for cytopathic effects. Isolates were identified by indirect fluorescent antibody test (IFAT) using serotype-specific monoclonal antibodies [34] and infected supernatant was clarified by centrifugation and virus stocks stored in 1-mL aliquots at −70°C.

### RNA extraction

Viral RNA was extracted using QIAamp Viral RNA Mini kit (Qiagen) following the manufacturer’s instructions and stored at -70°C for DENV typing and sequencing. For the viral 3′UTR sequencing, the RNA was extracted directly from serum and mosquitoes macerate, previously detected by RT-PCR. For the full genome sequencing of the DENV-3 strain BR73354/01, the RNA was extracted from the first passage in cell culture.

### RT –PCR (Reverse transcriptase- polymerase chain reaction)

RT—PCR for detecting and typing DENV was performed as described previously [35].

### Sequencing and phylogenetic analysis

PCR products were sequenced in both directions using the BigDye Dideoxy Terminator sequencing kit (Applied Biosystems). The mosquitoes’ DENV-3 full-length genome sequence and 3′UTR sequences obtained in this study were deposited in GenBank ( http://www.ncbi.nlm.nih.gov) and are described on Table 1. Sequence and similarity identity analysis was performed using BioEdit software ( http://www.mbio.ncsu.edu/bioedit/bioedit.htmL). The multiple alignments were performed using CLUSTAL W ( http://www.ebi.ac.uk/clustalw/) and the phylogenetic analysis by MEGA 5 software ( http://www.megasoftware.net), using the Neighbor-joining method, according to the Tamura-Nei model, with a bootstrap of 1,000 replications for the analysis of the complete genome. For the 3′UTR analysis, the Maximum likelihood method, according to the Kimura-2 model was chosen as determined by the best-fit substitution model provided by MEGA 5. Strains representative from the five genotypes available in GenBank ( http://www.ncbi.nlm.nih.gov) were used for the comparison, DENV-1 (GenBank accession number #AF513110), DENV-2 (#AF489932) and DENV-4 (# AF326573) strains were used as outgroup to root the tree.

### Secondary structure analysis

The predicted secondary structures were generated by MFOLD web server ( http://mfold.rna.albany.edu/?q=mfold/RNA-Folding-Form) with default folding parameters and folding predictions at 37^o^C on the VR from the 3’ UTR of DENV-3.

## Results and discussion

The DENV-3 was re-introduced in Latin America in 1994, after an absence of 17 years, being initially isolated in Panama and spreading throughout Central America [36,37] to Caribbean [38-40] and South America [6,28,41-43]. However, some phylogenetic studies point to its introduction through Mexico [44] a few years earlier [13]. This introduction caused by the genotype III of DENV-3, originally from the Southeast Asia and characterized by an increased virulence, coincided with the occurrence of a higher number of severe and DHF cases [37,45-47].

In Brazil, the first DENV-3 was isolated in December of 2000 in Nova Iguaçu, RJ [5] when the *Ae aegypti* infestation level was 8.1% and, 58% of those mosquitoes were resistant to temephos at that time [8,48]. Due to the role of the city of Nova Iguaçu in dengue epidemiology, after the DENV-1 introduction in 1986 [4], field studies were conducted for detection of DENV in field-caught vectors [8,49]. The potential emergence of strains associated with severe disease highlights the need for the surveillance of DENV in human host and vectors, as the detection of DENV in infected field-caught vectors is considered a useful tool for the early prediction of epidemics and detection of new serotypes/genotypes introductions [50,51].

The entomological surveillance performed in the first semester of 2001 in Nova Iguaçu, RJ, resulted in the isolation of three DENV-3 strains from the districts of Santa Efigênia (BR73354/01), California (BR73356/01) and Morro Agudo (BR73636/01) isolated from three pools (9 mosquitoes/pool) of naturally infected *Ae. aegypti* females [8]. In January 2006, one DENV-3 strain (BR81200/06) was isolated from one *Ae. aegypti* pool composed of three females, collected indoors in the Vargem Pequena neighborhood, west region of RJ.

**Figure 1:**
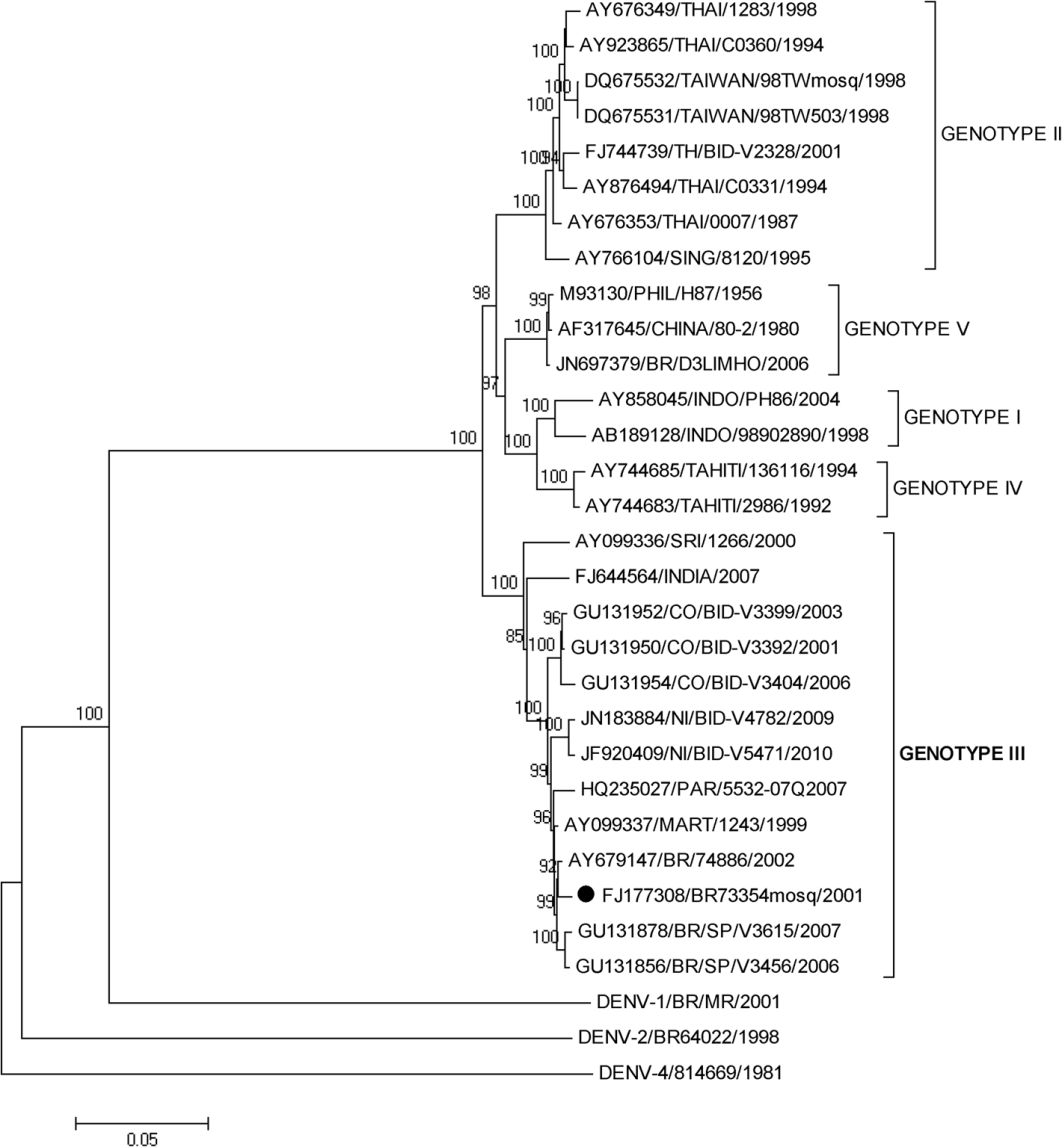
**Neighbor-joining phylogenetic analysis of the complete genome sequence from DENV-3 isolated from naturally infected mosquitoes in Brazil, 2001.** Black circle represent DENV-3 sequence generated in this study. Strains representative from the four genotypes available in Genbank ( http://www.ncbi.nlm.nih.gov) were used for the comparison, DENV-1, DENV-2 and DENV-4 strains were used as outgroup to root the tree. The percentage of replicate trees in which the associated taxa clustered together in the bootstrap test (1000 replicates) is shown next to the branches. DENV strains used were named as follows: GenBank accession number/country/year

In order to access the differences among Brazilian DENV-3, we sequenced and deposited on Genbank (FJ177308) the entire genome sequence of one virus isolated from naturally infected *Ae. aegypti* (BR73354/01) and compared to the Brazilian strain 74886/02 (AY679147), isolated from the liver of a fatal case during the DENV-3 epidemic occurred in 2002 [31]. The nucleotide similarity was 99.3% and the phylogeny based on the analysis of the complete coding region characterized the Brazilian strain as belonging to genotype III (Indian Subcontinent), Figure 1.

Amino acid substitutions were observed throughout the entire coding region, when the Brazilian DENV-3 strains were compared to the prototype PHIL/H87/1956 and strains representative of the other genotypes. Some substitutions were exclusive to the Brazilian DENV-3 strains analyzed (Table 2) and some were shared among the Brazilian strains and the strain isolated from *Ae. aegypti* in Taiwan in 1998 (TAIWAN/TWmosq/1998), Table 2. Exclusive substitutions to the DENV-3 Brazilian strains analyzed in this study were observed on the capsid, prM and envelope genes. Besides those others observed were also shared by other genotype III strains previously analyzed [11].

**Table 2:** Amino acid differences among selected Brazilian DENV-3 and strains representative of the DENV-3 genotypes, compared to the prototype H87

**Genotypes**	**GI**			**GII**			**GI**	**GII**	**GIV**
**Gene**	**Strain**	**PHIL/H87/1956 human**	**BR/74886/ 2002 human**	**BR/73354/2001 mosquitoes**	**BR/SP/V3456/2006 human**	**BR/SP/V3615/2007 human**	**INDO/PG86/2004 human**	**TAIWAN/TWmosq/ 1998 mosquitoes**	**TAHITI/136116/1994 human**
	**Position**								
Capsid	C _108_	M	**I**	**I**	**I**	**I**			
	C_112_	T	**A**	**A**	**A**	**A**			
prMembrane	prM_86_	H	**R**	**R**	**R**	**R**			
Envelope	E_81_	I	**V**	**V**	**V**	**V**			
	E_124_	S	**P**	**P**	L	L	L	**P**	
	E_132_	H	**Y**	**Y**	**Y**	**Y**		**Y**	
	E_169_	A	**T**	**T**	**T**	**T**	V	V	V
	E_270_	T	**N**	**N**	**N**	**N**		**N**	
	E_301_	L	**T**	**T**	**T**	**T**	S		
	E_380_	I			**T**	**T**			
	E_383_	K		**N**	**N**	**N**			
	E_452_	I	**V**	**V**	**V**	**V**			
NS1	NS1_84_	I			**V**				
	NS1_94_	T	I		I	I	I	I	I
	NS1_139_	S	N		N	N	N	N	N
	NS1_339_	N	**S**	**S**	**S**	**S**			
NS2A	NS2A_37_	L	**F**	**F**	**F**	**F**			
	NS2A_158_	I	**M**	**M**	**M**	**M**			
	NS2A_175_	I	**V**	**V**	**V**	**V**		**V**	
	NS2A_195_	T	**A**	**A**	**A**	**A**			
NS2B	NS2B_60_	V	**I**	**I**	**I**	**I**			
	NS2B_109_	I	**V**	**V**	**V**	**V**			
NS3	NS3_115_	I	**T**	**T**	**T**	**T**			
	NS3_452_	V	**A**	**A**	**A**	**A**		**A**	
NS4A	NS4A_99_	D	**E**	**E**	**E**	**A**			
NS4B	NS4B_21_	V	**I**	**I**	**I**	**I**			
	NS4B_138_	V	**I**	**I**	**I**	**I**			
NS5	NS5_97_	V		**L**					
	NS5_98_	T		**S**					
	NS5_101_	R		*STOP					
	NS5_105_	K		**T**					
	NS5_112_	E		**A**					
	NS5_114_	V		**R**					
	NS5_117_	S		**P**					
	NS5_127_	M		**R**					
	NS5_131_	D		**E**					
	NS5_133_	F		**D**					
	NS5_135_	L		**P**					
	NS5_229_	S	**A**	**A**	**A**	**A**			
	NS5_233_	M		**R**					
	NS5_288_	S	**N**	**N**	**N**	**N**			
	NS5_365_	P	**S**	**S**	**S**	**S**			
	NS5_371_	K	**R**	**R**	**R**	**R**			
	NS5_374_	E	**G**	**G**	**G**	**G**			
	NS5_389_	R		**K**	**K**	**K**		**K**	
	NS5_422_	R	**K**	**K**	**K**	**K**			
	NS5_429_	E	**D**	**D**	**D**	**D**			
	NS5_585_	K	**T**	**T**	**T**	**T**			
	NS5_639_	L	**P**	**P**	**P**	**P**			
	NS5_763_	T	**S**	**S**	**S**	**S**	A	S	A
	NS5_835_	D **N**	**N**	**N**	**N**				

Blank cells indicate amino acid similarities to the prototype strain H87 (GenBank accession number M93130). *STOP: stop codon.

On the NS1 gene only one residue substitution was observed, and substitution on NS2A and NS2B were also reported. On the NS3 gene, a substitution on only NS3_115_ was exclusive to the Brazilian DENV-3. On NS4B, the same substitution was observed on positions NS4B_21_ and NS4B_138_. However, on the NS5 gene, besides the ten substitutions exclusive to the Brazilian DENV-3, substitutions exclusive to the Brazilian DENV-3 isolated from *Ae. agypti* (BR73354/2001) were observed almost consecutively, on NS5_97_ and NS5_98_. On NS5_101_, the substitution resulted in a stop codon and other substitutions throughout NS5 were also observed. Despite this, the original macerate was re-inoculated in C6/36 cells culture and the DENV −3 infection confirmed by the presence of cytopathic effect and a positive RT-PCR. The presence of genome-defective DENV-3 containing either stop codons or deletions *in vivo* has been reported previously [52].

Despite the use of the E gene for DENV phylogenetic and evolutionary studies [9,12,28,53-62] due to its biological properties and selective pressure imposed by the host immune response, the role of sequences heterogeneity in other genomic regions which includes the non-structural genes and the genome UTRs cannot be excluded [53].

Previous studies have suggested that the sequence and secondary structures of the 5′ and 3′UTR of flaviviruses play an important role in viral replication and differences in these regions may influence viral virulence [24,26,27,63]. Mutations and deletions within these regions may alter infectivity and reduce efficiency of viral replication [22,23]. Furthermore, the domain I from the DENV 3′UTR is considered the most variable region (VR) from the 3′UTR [18] and can serve as a good marker for DENV evolution [19-21].

The analysis of the 3′UTR of the strain BR73354/01 genome showed an 8- nucleotides deletion within the 11-nucleotides insertion on the VR, previously observed for the Brazilian DENV-3 strain isolated from humans [31] and common to genotype III DENV-3 strains from the Latin America/Caribbean and Sri Lanka regions [27,64]. Nucleotides substitutions exclusive to the BR73354/01 were observed on positions 10,383 and 10,391 from the 3′UTR. One substitution on the RCS2 and one on the CS2 were shared by all Brazilian DENV-3 when compared to the prototype.

**Figure 2:**
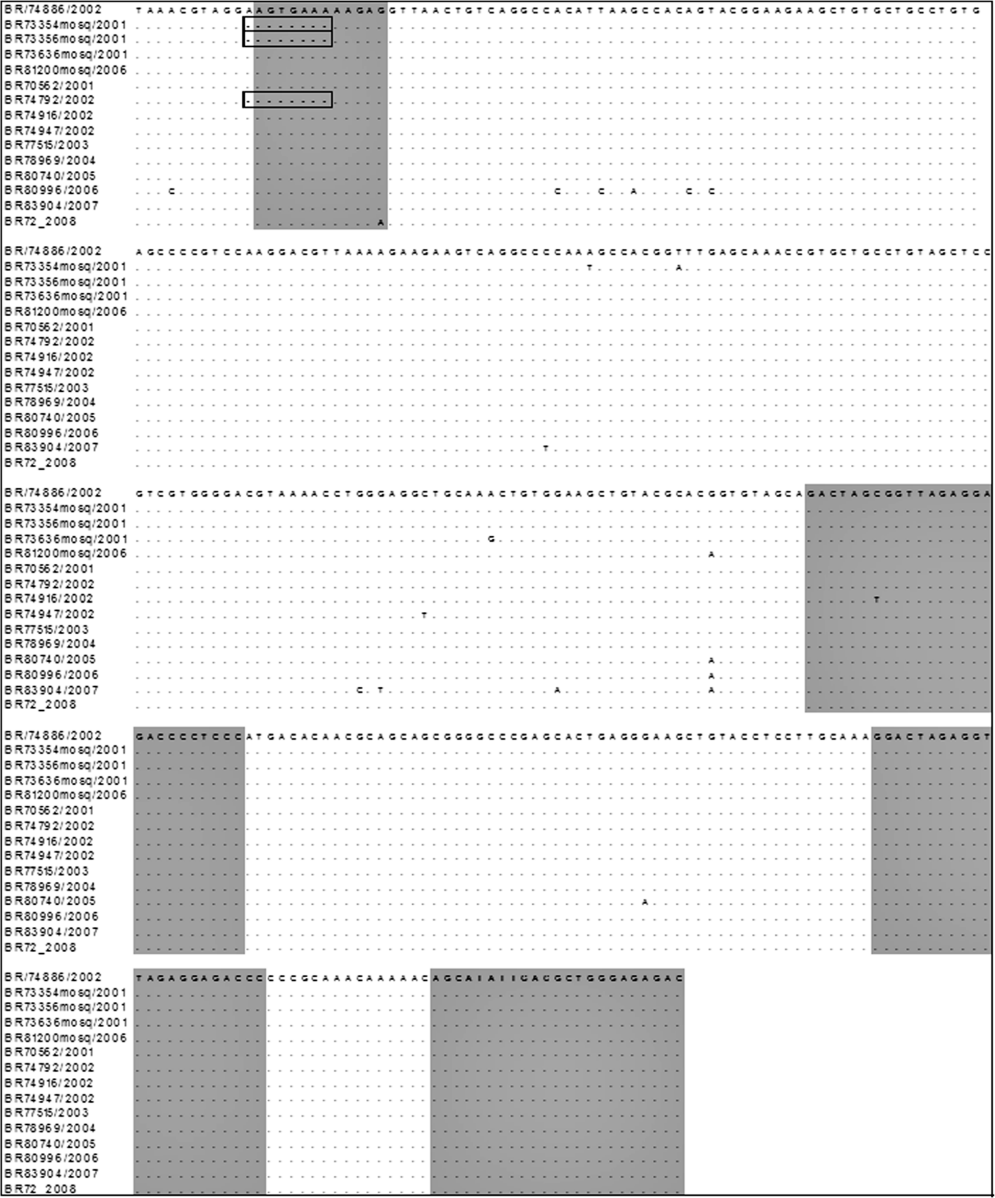
**Multiple nucleotide sequence alignment of the Brazilian DENV-3 3′UTR from additional strains isolated from mosquitoes *Ae. aegypti *(*n=*4) and humans (*n=*10) from 2001 to 2008.** Dots (.) indicate identity among strains based on the Brazilian strain BR74886/02, characterized by the 11 nucleotides insertion on the variable region (VR). Dashes (−) indicate gaps in the alignment. The 8 nucleotides deletion characteristic to strains isolated from mosquitoes BR73354mosq/2001, BR73356mosq/2001 and from a strain isolated in human (BR74792/2002) are marked by black squares. Eleven nucleotide insertion and conserved sequence regions (RCS2, CS2 and CS1) are gray-shadowed

**Figure 3:**
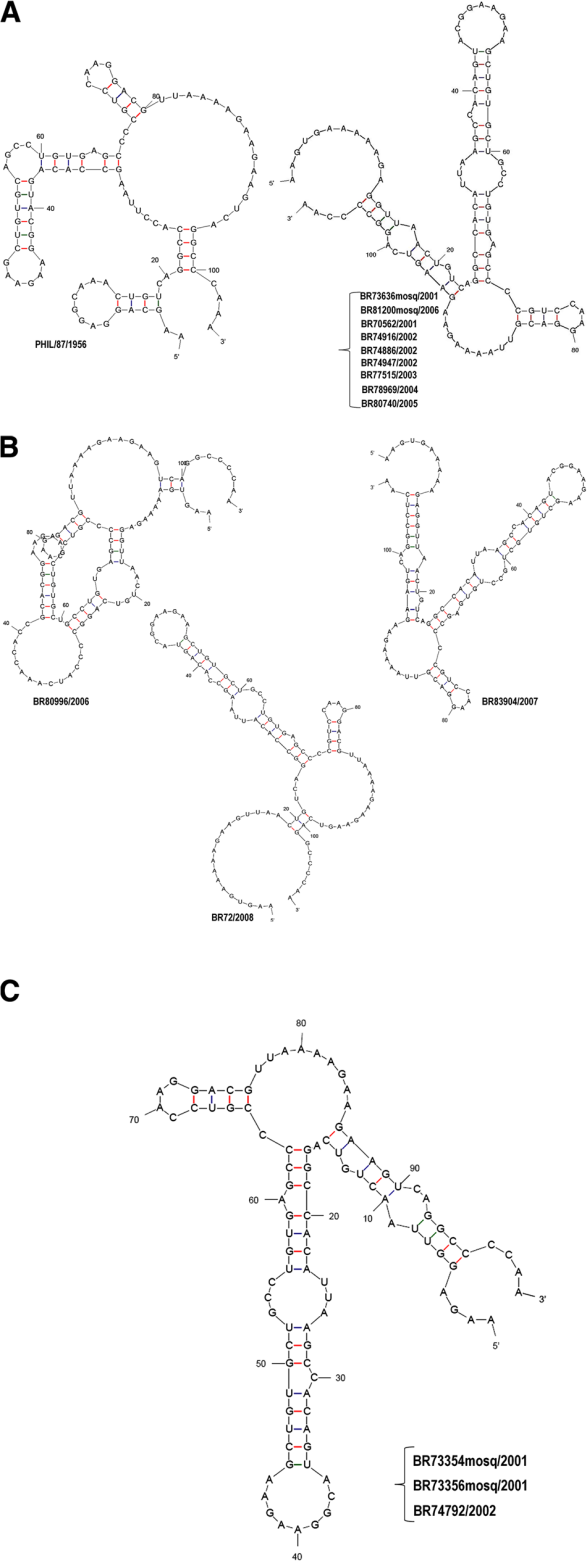
**On (A) the predicted secondary structure of the variable region (VR) from Brazilian DENV-3 strains isolated from humans and mosquitoes *Ae. Aegypti *(nucleotides 1 to 108) presenting the 11-nucleotides insertion and compared to the prototype PHIL/87/1956 (nucleotides 1–104).** On (**B**) the predicted secondary structures from the Brazilian strains (nucleotides 1 to108) presenting the 11-nucleotides insertion, but with nucleotides substitutions differing from the strains on (**A**) and on (**C**) the structures from the sequences (nucleotides 1 to 100) with the 8-nucleotides deletion

We additionally analyzed and deposited on GenBank the 3′UTR sequences from other three DENV-3 strains isolated from naturally infected *Ae. aegypti* isolated in 2001 and 2006 in RJ (BR73356/01, BR73636/01 and BR81200/06) and from ten DENV-3 isolated from humans from 2001 to 2008. The strain BR73356/01 presented the same 8-nucleotides deletion observed for the strain BR73354/01. However, the other two strains also isolated from mosquitoes in RJ (BR73636/01 and BR81200/06) presented the 11-nucleotides insertion common to the human strains. The analysis of the 3′UTR from DENV-3 isolates from humans showed that nine out of ten strains also presented the 11-nucleotides previously described. However, one of the strains isolated in Rio de Janeiro in 2002 (BR74792/02) showed the same 8-nucleotides deletion observed on the mosquito strains (Figure 2). Previous studies have shown deletions and nucleotide variations in the VR within a same serotype [28,29]. Despite those observations, it was also shown a high conservation on the 3′UTR RCS2, CS1 and CS2 conserved regions (Figure 2, gray-shadowed areas) among all the Brazilian strains analyzed. This was quite expected as domain II is of moderate conservation and domain III, the most conserved region of all [18].Therefore, we focused on the VR of the 3′UTR, aiming to better characterize those mutations by predicting the secondary structures of that region. Not all strains presenting the 11-nucleotides insertion were similar in structure (Figure 3A and 3B). In fact, despite the 11-nucleotides insertion, the strain BR72/2008 presented a unique secondary structure (Figure 3B). The only difference from the latter is a nucleotide substitution (G →A) on the 11-nucleotides insertion region, when compared to all other Brazilian sequences analyzed (Figure 2). The strain BR80996/2006, also showed a unique secondary structure due to the nucleotides substitution presented in the VR, despite the presence of the insertion shared with the other strains. The slight difference presented by the strain BR83904/2007, was due to a substitution (C→T) exclusive to this sequence. Furthermore, all three  sequences with  the  8-nucleotides  deletion  (BR73354mosq/2001, BR73356mosq/2001 and BR74792/2002) presented the same secondary structure (Figure 3C).

Phylogenetic studies based on the 5′and 3′UTR have shown to be very useful for molecular epidemiological studies [19,21,27,28,65]. The Maximum-Likelihood phylogenetic tree of Brazilian DENV-3 strains isolates from naturally infected *Ae. aegypti* mosquitoes and humans based on the 3′UTR sequence analysis places those strains as belonging to genotype III, corroborating the findings of the full-length genome analysis (data not shown).

## Conclusions

Here, we analyzed the coding region and the 3′UTR of DENV-3 from both human host and mosquitoes and described insertions, deletions and a substitution leading to stop codon formation. The majority of DENV-3 in this study was characterized by the 11-nucleotide insertion in the 3′UTR, despite the observation of strains carrying the 8-nucleotide deletion. In spite the presence of distinct viral variants, it is suggested that the major variant is transmitted. However, how those distinct viral populations are maintained or transmitted is not fully understood, therefore the availability of viruses isolated from both hosts are crucial for the better comprehension of the vector-virus-human host interactions and for quasispecies investigations. Furthermore, the analysis of those distinct viral populations in experimentally infected mosquitoes may help to elucidate those observations.

## Competing interest

The authors have no conflict of interest.

## Authors’ contributions

FBS, RMRN and RLO designed the study. MGC, FNB performed the experiments, MGC, FBS and RLO wrote the paper. All authors read and approved the final manuscript.

## Financial support

CNPq, CAPES, FIOCRUZ, FIOCRUZ/PAPES V and FAPERJ. The funders had no role in study design, data collection and analysis, decision to publish, or preparation of the manuscript.
